# Interlaboratory Comparison of a Biomimetic Extraction Method Applied to Oil Sands Process–Affected Waters

**DOI:** 10.1002/etc.5340

**Published:** 2022-06-25

**Authors:** Daniel J. Letinski, Asfaw Bekele, Martin J. Connelly

**Affiliations:** ^1^ Health & Environmental Applications Division, ExxonMobil Biomedical Sciences Annandale New Jersey USA; ^2^ Upstream Research, Imperial Calgary Alberta Canada

**Keywords:** Solid‐phase microextraction, Biomimetic extraction, Oil sands process–affected waters

## Abstract

Biomimetic extraction using solid‐phase microextraction is a passive sampling analytical method that can predict the aquatic toxicity of complex petroleum substances. The method provides a nonanimal alternative to traditional bioassays with the potential to reduce both vertebrate and invertebrate aquatic toxicity testing. The technique uses commercially available polydimethylsiloxane‐coated fibers that, following nondepletive extraction of water samples, are injected into a gas chromatograph with flame ionization detection. As the predictive nature of the method is operationally defined, it is critical that its application be harmonized with regard to extraction, analysis, and standardization parameters. Results are presented from a round robin program comparing the results from 10 laboratories analyzing four different sample sets of dissolved organics in water. Samples included two incurred oil sands process–affected waters and a cracked gas oil water accommodated fraction. A fourth sample of cracked gas oil blended in an oil sands process–affected water was analyzed to demonstrate the method's ability to differentiate between neutral and ionizable dissolved hydrocarbons. Six of the 10 laboratories applied an automated version of the method using a robotic autosampler where the critical extraction steps are precisely controlled and which permits batch screening of water samples for aquatic toxicity potential. The remaining four laboratories performed the solid‐phase microextraction manually. The automated method demonstrated good reproducibility with between‐laboratory variability across the six laboratories and four samples yielding a mean relative standard deviation of 14%. The corresponding between‐laboratory variability across the four laboratories applying the manual extraction was 53%, demonstrating the importance of precisely controlling the extraction procedure. *Environ Toxicol Chem* 2022;41:1613–1622. © 2022 The Authors. *Environmental Toxicology and Chemistry* published by Wiley Periodicals LLC on behalf of SETAC.

## INTRODUCTION

Biomimetic extraction using solid‐phase microextraction (BE‐SPME) is a passive sampling analytical method that can predict the acute aquatic toxicity of complex petroleum substances by measuring the bioavailability of dissolved hydrocarbons in water. It can also serve as an exposure metric for aquatic toxicity testing (Leslie et al., 2002; Parkerton et al., 2000). The method was developed as a tool to estimate total‐body residues in biota after exposure to organic chemicals (van Loon et al., 1996). It is based on the principle that polydimethylsiloxane (PDMS)–water partition coefficients correlate well with octanol–water and membrane–water partition coefficients, with the fiber coating serving as a surrogate for lipid partitioning (Verbruggen et al., 2000). Hydrocarbons partition into the PDMS coating in proportion to their freely dissolved concentrations and are driven by the partitioning properties of the individual constituents. This parallels the mechanistic basis used to predict aquatic toxicity in the PETROTOX model and thus supports both the model and the premise proposed by Redman, Butler, et al. (2018). Because the SPME extractions are performed in a nondepletive manner, hydrocarbons that partition into the SPME fiber represent the bioavailable fraction of the hydrocarbon exposure. Nondepletive, or negligible depletion, SPME has been variously defined by researchers as a reduction of as little as 1% (Górecki & Pawliszyn, 1997) to as much as 5% (Vaes et al., 1996) or 10% (Parkerton et al., 2000; Poerschmann et al., 1997) of the original sample concentration of dissolved hydrocarbons. Non(negligible)depletion is most easily assessed experimentally by simply repeating the SPME extraction on a previously extracted sample to confirm that the measured on‐fiber amounts are constant. The key to establishing nondepletive extraction conditions is to employ a low PDMS to water ratio (Redman, Butler, et al., 2018). The BE‐SPME method has the potential to simplify aquatic hazard assessments of petroleum substances and aid in establishing water quality criteria in receiving waters because the total moles of hydrocarbons sorbed into the fiber can be related to toxicity thresholds in target lipid of aquatic organisms (Hedgpeth et al., 2019).

The BE‐SPME method has been most frequently applied to the assessment of the aquatic toxicity hazard of complex petroleum substances including crude oil (Bera et al., 2018; Letinski et al., 2014; Redman & Parkerton, 2015; Redman et al., 2017), refined products (CONCAWE, 2016; Redman et al., 2014), and petrochemicals (Woods et al., 2007). Also, SPME‐based biomimetic extractions have been used to assess both the aquatic toxicity and bioaccumulation potential of refinery effluents by measuring the bioavailability of hydrocarbons in these waters (Cailleaud et al., 2019; Comber et al., 2015; Leonards et al., 2011; Whale et al., 2022; Worden et al., 2021). A majority of published BE‐SPME applications have focused on capturing dissolved neutral organics. The technique was extended to dissolved ionizable organics associated with a commercial naphthenic acid product by acidifying aqueous samples prior to SPME extraction and demonstrated biomimetic extraction of dissolved ionizable organics to be consistent with a nonpolar narcosis mode of toxicity (Swigert et al., 2015). For dissolved ionic organics (i.e., naphthenic acids), the molecules are protonated following sample acidification and partition as their neutral form into the PDMS polymer. The application of the acid‐modified BE‐SPME method was significantly extended as an exposure metric in assessing the aquatic toxicity of a series of individual organic and naphthenic acids, defined mixtures, and acid extracts of organics derived from oil sands process–affected waters (OSPW) sourced from the development of oil sands resources in northern Alberta, Canada (Redman, Parkerton, et al., 2018). More broadly, the ability of BE‐SPME to predict aquatic toxicity of both dissolved neutral and ionizable organics provides a nonanimal alternative to traditional bioassays offering the potential to reduce both vertebrate and invertebrate aquatic testing (Norberg‐King et al., 2018; Scholz et al., 2013). The method also allows for greater sample throughput in screening for aquatic toxicity potential because the entire extraction and analysis cycle is completed in approximately 2 h for each sample compared to traditional bioassays, which generally span 48–96 h, depending on the test organism. Further efficiencies can be realized by the utilization of SPME autosamplers to accommodate batch sample screening. In addition, SPME has been used as a means to rapidly assess the efficacy of remediation treatments, such as ozonation, for the removal of bioavailable dissolved ionic organics from raw OSPW (Huang et al., 2020).

The BE‐SPME method utilizes commercially available, reusable SPME fibers coated with a PDMS polymer. A single fiber can be used for up to 100 injections and ultimately fails because of mechanical stress on the assembly's metal sheath or plunger, not because of degradation of the polymer coating. Following equilibration with water samples, the SPME fiber is thermally desorbed in the injection port of a gas chromatograph equipped with a flame ionization detector (GC‐FID). Quantification of the total moles of hydrocarbons on the fiber is accomplished by relating the total integrated area from the fiber to the response of a calibration surrogate standard: 2,3‐dimethylnaphthalene. The 2,3‐dimethylnaphthalene standard response is derived from a series of solvent‐based injections. This provides a direct GC on‐column response comparison of the total integrated area from the fiber to the standard. 2,3‐Dimethylnaphthalene was selected as the calibration standard during development of the method because it represents an alkylated diaromatic hydrocarbon that elutes at the approximate midpoint of fuel oils that were initially used to demonstrate the relationship between passive biomimetic extractions and aquatic toxicity.

Because BE‐SPME is operationally defined, it is critical that method parameters be harmonized and reproducible across laboratories to permit accurate interlaboratory comparison of results. The present study reports a harmonized BE‐SPME method including details of sample treatment, extraction, analysis, quantification, standardization, and reporting. It presents results from a round robin program involving 10 laboratories across four sample sets. The samples included two incurred OSPWs containing dissolved ionizable organics, primarily in the form of naphthenic acids. A third sample containing only dissolved neutral organics from a cracked gas oil water accommodated fraction (CGO WAF) was also evaluated. A fourth sample contained a blend of neutral and ionizable hydrocarbons to demonstrate the method's ability to differentiate by acidification of the sample prior to extraction. Six of the 10 laboratories applied an automated version of the method using a robotic autosampler where the critical extraction steps are precisely controlled, while the remaining four laboratories performed the SPME manually. Application of the automated versus manual technique was solely determined by each of the participating laboratories based on the availability of the necessary SPME autosampler at the time of the study. No predetermination or randomized selection was made with regard to this variable.

## MATERIALS AND METHODS

### Round robin program

Fourteen laboratories expressed preliminary interest in participating in the BE‐SPME OSPW round robin program. The laboratories represented a range of sectors including the petroleum industry, academic research, commercial environmental testing, and Canadian provincial and national governments. Representatives from each of the laboratories participated in an initial series of conference calls and webinars during which they were educated on the SPME technique and familiarized with the specific BE‐SPME application. Each laboratory was provided a protocol detailing the acid‐modified BE‐SPME GC‐FID method for analyzing OSPWs, which was discussed during the calls and in follow‐up communications, as needed. Replicates of a single field‐sourced OSPW sample were then supplied to each of the initial 14 participating laboratories. The purpose of this sample was to permit the laboratories to demonstrate basic proficiency in applying the BE‐SPME technique and to troubleshoot the method and optimize their instrumentation, as necessary. In addition, analysis of the preliminary OSPW sample and reporting of results in good order demonstrated the respective laboratories' willingness and commitment to establish the BE‐SPME capability and perform the method following the prescribed protocol. It also qualified the laborattoriess to participate in the more comprehensive round of testing across four sample sets, the results of which are reported in the present study. Ultimately, 10 laboratories qualified to participate in the comprehensive round robin program.

### Round robin samples

Four sets of samples were supplied to each of the 10 laboratories. The samples were identified by unique sample codes, and each set consisted of a total volume of approximately 180 ml distributed across three 60‐ml amber glass vials with no headspace and sealed with Teflon‐lined septa screw caps. The laboratories were instructed to keep samples refrigerated (~4 °C) pending SPME and analysis. The test sample identifications and descriptions are listed in Table 1. The OSPW samples were obtained from member companies of Canada's Oil Sands Innovation Alliance (COSIA), which represents the major operators developing oil sands resources in Alberta, Canada. Prior to shipment to the participating laboratories, bulk OSPW samples were allowed to settle and coarsely filtered to remove the potentially confounding influence of sorption to suspended matter. The CGO WAF was prepared using an intermediate catalytic cracked distillate (Chemical Abstracts Service [CAS] no. 64741‐60‐2) originally sourced from Canada Imperial Oil, an affiliate of Exxon Mobil (Calgary, AB, Canada). The CGO WAF and CGO blended in OPSW were prepared using a conventional WAF preparation technique. WAFs are a long‐accepted standard means of generating solutions of dissolved hydrocarbons of complex petroleum mixtures for aquatic hazard assessment (Aurand & Coelho, 1996; Coelho & Aurand, 1997; Girling et al., 1994; Singer et al., 2000; Wheeler et al., 2020). Briefly, 4 L of distilled water or OSPW was added to glass aspirator bottles, each sterilized with the equivalent of 50 mg/L mercuric chloride (HgCl_2_) to eliminate potential biodegradation during the WAF equilibration and sample storage. The CGO was added at a loading of 50 mg/L, and the WAFs were mixed for 48 h with a 10% vortex depth. Mixing was stopped 1 h prior to sampling. Individual replicates were randomly selected among the four sample types for inclusion in the sample sets sent to each participating laboratory.

**Table 1:** Round robin test samples and descriptions

Sample ID	Source/description	Constituents
June 2019 OSPW	Field sample	Naphthenic acids
July 2019 OSPW	Field sample	Naphthenic acids
CGO WAF	50 mg/L CGO WAF	Neutral hydrocarbons
CGO in OSPW	50 mg/L CGO WAF in composite OSPW	Naphthenic acids and neutral hydrocarbons

CGO WAF = cracked gas oil water‐accommodated fraction; OSPW = oil sands process–affected waters.

### BE‐SPME method

A complete protocol describing the BE‐SPME method is provided in the Supporting Information. Briefly, approximately 20‐ml water samples in sealed glass vials with septum caps were equilibrated with a 30‐µm PDMS SPME fiber (Millipore Sigma–Supelco) for 100 min with orbital agitation at 250 rpm at 30 °C on a robotic autosampler. Alternatively, where a suitable autosampler was not available, SPMEs were performed manually using a micro‐stir‐bar and stir‐plate by exposing the SPME fiber to the sample for 100 min with rapid stirring. Samples extracted at approximately neutral pH without acidification yield only dissolved neutral hydrocarbons. Samples acidified with 50 µl of phosphoric acid (H_3_PO_4_, 85%, American Chemical Society grade) to a pH of approximately 2 capture both neutral and polar, ionizable (acid) dissolved hydrocarbons. Following sample–fiber equilibration, the fiber is injected into the heated injection port of a GC‐FID, where the fiber is thermally desorbed for 3 min. Ideally, the GC is equipped with a narrow (≤1 mm inner diameter [i.d.]) injection liner and a 15 m‐wide‐bore, nonpolar capillary column. The total chromatographic peak area is attributable to compounds desorbed from the SPME fiber. Quantification of the total moles of hydrocarbons on the fiber is accomplished by relating the total integrated area from the fiber to the response of a calibration surrogate standard: 2,3‐dimethylnaphthalene. The 2,3‐dimethylnaphthalene standard response is derived from a series of solvent‐based injections. The method reporting units are expressed as equivalent molar concentrations of 2,3‐dimethylnaphthalene per volume (milliliters) of PDMS. The 10 laboratories were requested to analyze each sample set both with and without acidification, in duplicate, for each sample pretreatment. The specific extraction and analysis parameters applied by each laboratory are listed in Supporting Information, Table S1.

## RESULTS

**Figure 1:**
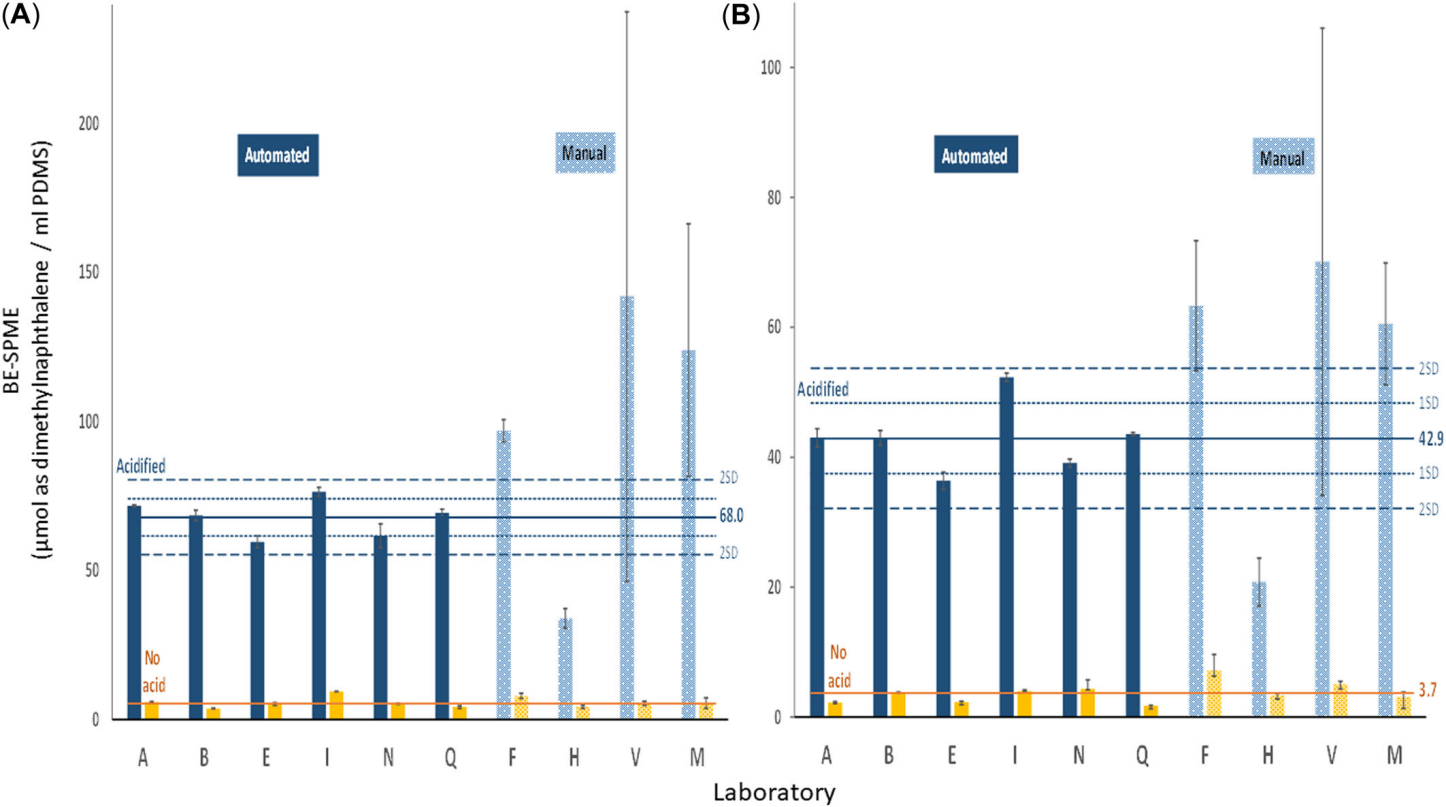
Interlaboratory comparison of biomimetic extraction using solid‐phase microextraction results—oil sands process‐affected waters samples (**A**) June 2019, (**B**) July 2019. Solid bars represent results measured using the automated method. Hashed bars represent results measured using the manual method. Blue bars represent acidified samples. Yellow bars represent nonacidified samples. Means (solid lines), 1 standard deviation (dotted lines), and 2 standard deviations (dashed lines) include only results for acidified samples from laboratories applying the automated method. For nonacidified samples, only the mean (solid line) is presented. BE‐SPME = biomimetic extraction using solid‐phase microextraction; PDMS = polydimethylsiloxane.

**Figure 2:**
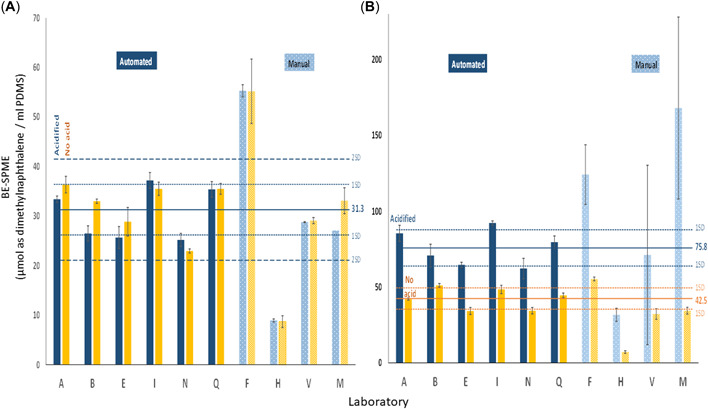
Interlaboratory comparison of biomimetic extraction using solid‐phase microextraction results—cracked gas oil samples. Solid bars represent results measured using the automated method. Hashed bars represent results measured using the manual method. Blue bars represent acidified samples. Yellow bars represent nonacidified samples. (**A**) Cracked gas oil (50 mg/L) water‐accommodated fraction. Mean (solid lines), 1 standard deviations (dotted lines), and 2 standard deviations (dashed lines) calculated using combined results for both acidified and nonacidified samples from laboratories applying the automated method. (**B**) Cracked gas oil in oil sands process–affected waters. Mean (solid lines) and standard deviations (dotted lines) presented separately for both acidified and nonacidified samples from laboratories applying the automated method. BE‐SPME = biomimetic extraction using solid‐phase microextraction; PDMS = polydimethylsiloxane.

Except where specifically noted, the discussion that follows refers to BE‐SPME results of the acidified samples as this captures the total dissolved organics, both ionizable and neutral. For brevity, the BE‐SPME units of micromoles of 2,3‐dimethylnaphthalene per milliliter of PDMS are simply referred to as “BE units.” Results for the two OSPW samples are presented in Figure 1. Results for the two CGO samples are presented in Figure 2. The reported means and 1 and 2 standard deviations (SDs) on each plot are calculated based only on those laboratories applying the automated method (*n* = 6). Table 2 summarizes the results including measured variability both within and across laboratories and segregated by laboratories applying the automated versus manual technique. The results for the automated and manual procedures are reported separately because the variances from the two procedures are significantly different at the 5% level of statistical significance, according to Levene's test of equality of variances. Additional information related to the test for the equality of variances from the two methods is provided in Supporting Information, Figure S1A,B. The overall mean variability across all 10 laboratories for the four sample sets was 38%. However, for the six laboratories applying the automated method the relative SD (RSD) was only 14% compared to 53% for the four laboratories applying a manual version of the BE‐SPME method.

**Table 2:** Summary of biomimetic extraction using solid‐phase microextraction interlaboratory results

	All laboratories (*n* = 10)		Automated (*n* = 6)		Manual (*n* = 4)	
Sample description	Acidified	Not acidified	Acidified	Not acidified	Acidified	Not acidified
OSPW June 2019						
Mean	80.5	5.71	68.0	5.64	99.3	5.80
SD	32.1	1.76	6.27	2.03	47.3	1.55
RSD (%)	40	31	9.2	36	48	27
OSPW July 2019						
Mean	47.2	3.7	42.9	3.04	53.7	4.63
SD	14.6	1.58	5.39	1.09	22.3	1.87
RSD (%)	31	43	13	36	42	40
50 mg/L CGO WAF						
Mean	30.4	31.8	30.6	32.0	30.0	31.5
SD	11.7	11.7	5.37	5.21	19.1	19.1
RSD (%)	39	37	18	16	64	60
50 mg/L CGO in OSPW						
Mean	85.0	38.4	75.8	42.5	98.8	32.3
SD	37.5	13.6	11.9	7.05	59.7	19.7
RSD (%)	44	35	16	17	60	61
Mean RSD across all samples (%)	38	36	14	26	53	47

BE‐SPME reporting units: micromoles as 2,3‐dimethylnaphthalene/ml polydimethylsiloxane.

OSPW = oil sands process–affected waters; SD = standard deviation; RSD = relative SD; CGO WAF = cracked gas oil water accommodated fraction.

Sample June 2019 OSPW (Figure 1A) yielded a mean of 68.0 BE units with an RSD of 9.2% across the six laboratories applying the automated method. The mean nonacidified value across all 10 laboratories was 5.7 BE units with an SD of 2.0. Sample July 2019 OSPW (Figure 1B) yielded a mean of 42.9 BE units with an RSD of 13% across the six laboratories applying the automated method. The mean nonacidified value across all 10 laboratories was 3.7 BE units with an SD of 1.6. For the two OSPW samples, the results reported by five of the six laboratories were within 1 SD, whereas the sixth laboratory was within 2 SDs, of the mean. For the two incurred OSPWs, the nonacidified (neutral dissolved organics) BE contribution is approximately 7.7% of the acidified (sum of ionizable and neutral) BE.

For the CGO WAF sample (Figure 2A), the mean BE was determined using the combined acidified and nonacidified results because acidification did not appear, nor was it expected, to have a significant impact on the recovery of the dissolved neutral hydrocarbons associated with this petroleum substance. The combined mean across the six laboratories applying the automated method was 31.3 BE units with an RSD of 16% for the gas oil sample.

The mean acidified BE‐SPME for the CGO in OSPW (Figure 2B) was 75.8 BE units with an RSD of 16% across the six laboratories applying the automated method. The corresponding nonacidified mean was 42.5 BE units with an RSD of 14%. Results from this sample demonstrate the ability of the BE‐SPME method to distinguish between significant amounts of ionizable and neutral dissolved organics in the same sample simply by altering the pH.

The magnitude of the error bars in Figures 1 and 2 also tends to be significantly larger for the laboratories applying the manual method, indicating greater intralaboratory variability across replicate sample analyses compared to the laboratories applying the automated technique.

**Figure 3:**
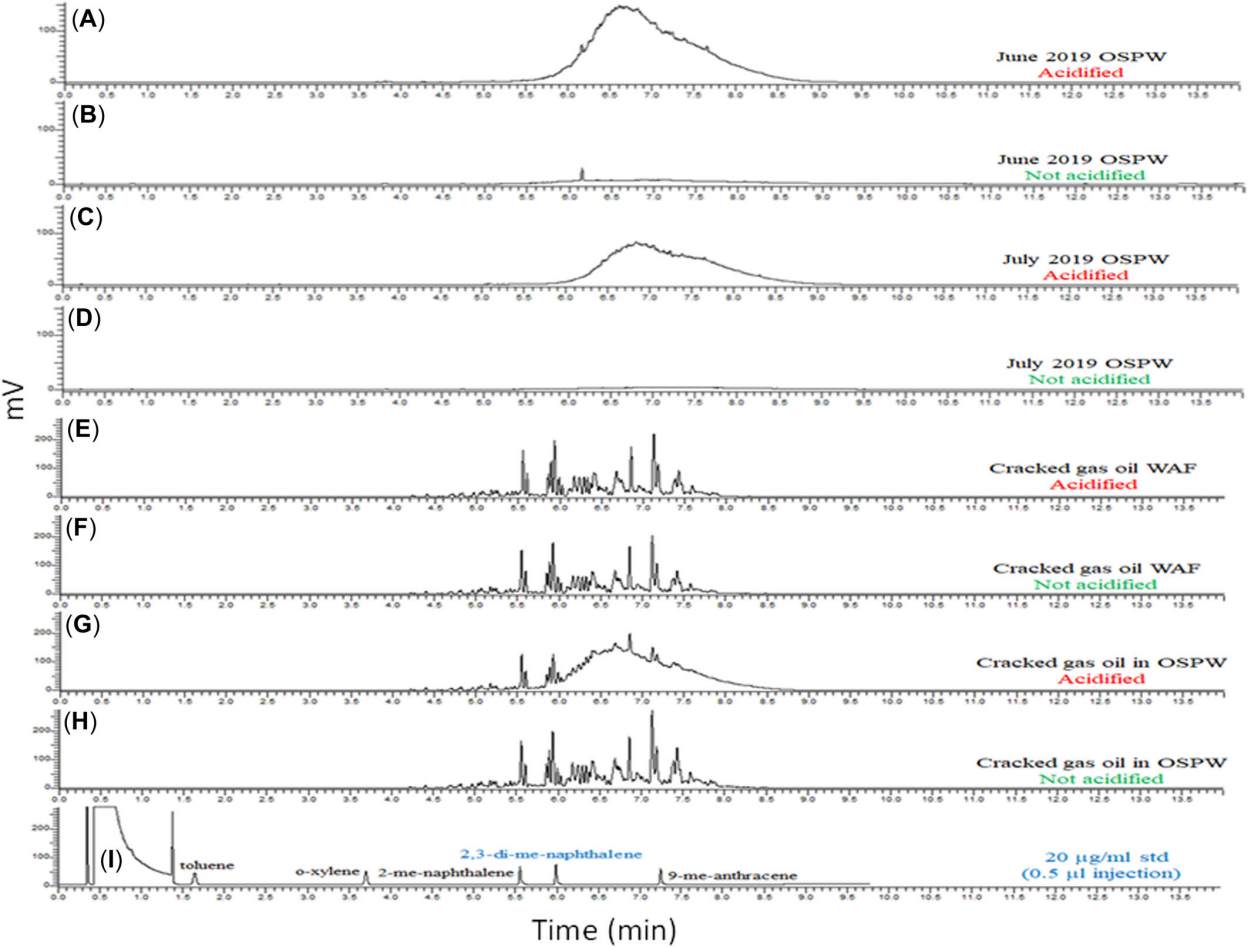
Biomimetic extraction using solid‐phase microextraction: gas chromatography equipped with a flame ionization detector chromatograms acquired using the automated method (30 μm polydimethylsiloxane, 100 min, 250 rpm orbital agitation). OSPW = oil sands process–affected waters; WAF = water accommodated fraction.

Representative SPME GC‐FID chromatograms are presented in Figure 3. These were generated by ExxonMobil Biomedical Sciences, which coordinated the round robin program and developed the BE‐SPME protocol. The two incurred OSPW samples, June 2019 OSPW and July 2019 OSPW, acidified prior to SPME, resulted in chromatograms with a large, unresolved response eluting between approximately 5.5 and 9 min (Figure 3A, C). Analysis of the corresponding nonacidified portions of the same OSPWs results in chromatograms (Figure 3B, D) that approached that of background levels, confirming that the contribution of neutral hydrocarbons in the OSPW samples was low. In contrast, analysis of both the acidified and nonacidified portions of the CGO WAF samples resulted in a series of discrete chromatographic peaks, each of similar magnitude, characteristic of refined petroleum mixtures and indicating, as expected, little in the way of ionizable or polar constituents (Figure 3E, F).

Chromatograms (Figure 3G, H) of the CGO in OSPW sample demonstrate the ability of the method to discriminate between the total ionizable and neutral dissolved organics (acidified) versus neutral only dissolved organics (nonacidified) sample.

**Figure 4:**
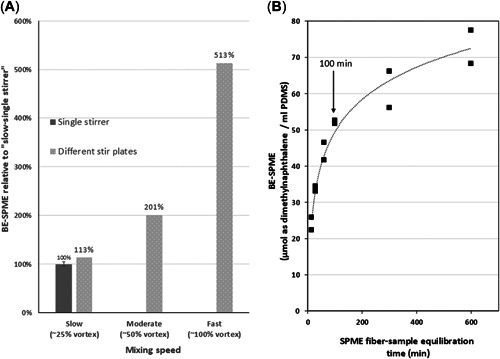
Effect of manual stirring and automated agitation kinetics of an oil sands process–affected waters sample using a 30‐μm polydimethylsiloxane solid‐phase microextraction (SPME) fiber. (**A**) Effect of manual stirring speed on relative response compared to that achieved using a 25% mixing vortex and using different stir plates and 100 min extraction time. (**B**) Measured on‐fiber SPME concentrations versus automated 250 rpm orbital agitation time. BE‐SPME = biomimetic extraction using solid‐phase microextraction; PDMS = polydimethylsiloxane.

Additional data were developed in an effort to explain the greater variability measured across laboratories applying the manual method. When applying the manual method, a small stir‐bar, of unspecified size and geometry, is added to the sample; and the mixing rate is less precisely controlled by the stir‐plate. Stir‐bar mixing at high rates tends to be more efficient than orbital agitation at reaching sample–fiber equilibrium. Separate work on ethyl octanoate and other ethyl esters in wine samples demonstrated that, in terms of maximized response attributable to SPME, sample stirring > orbital agitation > static equilibrium (GERSTEL, 2021). This is partly demonstrated as part of the present study using data provided by one of the participating laboratories using the manual method applied to the July 2019 OSPW acidified sample. Figure 4A shows the results of varying the stir‐bar mixing rate. The increase in mixing rate resulted in a 5‐fold increase in BE response, progressing from a slow stirring speed (vortex depth ~25% of vial height) up through moderate (50% vortex depth) and fast speeds (100% vortex depth). The laboratory also took care to expose the fiber in the vial approximately equidistant between the vial wall and the center of the vortex. Also contributing to the difference is the likelihood that ionizable organics associated with OSPW samples have not reached equilibrium with the SPME fiber after 100 min of orbital agitation. To confirm this, Figure 4B was generated by varying the automated SPME orbital agitation time from 15 to 600 min for analysis of an acidified OSPW sample. At 100 min, the measured BE value is approximately 70% of that reached after 600 min. Even after 600 min of orbital agitation, the sample constituents (i.e., naphthenic acids) still appear to have only reached a quasi‐equilibrium with the PDMS fiber. These factors likely explain the higher resultant BE values for the manually derived BE values using the higher‐speed stir‐bar mixing. In contrast, the CGO WAF sample yielded more similar mean BE values across the laboratories applying the automated and manual methods, though variability across the laboratories using the manual technique was greater. This implies that the dissolved neutral organics associated with the gas oil are likely closer to equilibrium than those components associated with the OSPWs after 100 min of equilibration. The SPME–water partition equilibrium and the associated chemical uptake are generally impeded by mass transfer from the water to the fiber, which is controlled by resistance in water boundary layers.

## DISCUSSION

The results using the automated BE‐SPME method yielded much lower inter‐ and intralaboratory variability relative to the results obtained using the manual method. The principal source of variability is likely attributable to the mixing rate and type during the SPME fiber–sample equilibration.

The results indicate that even with an equilibration time of 100 min, the entirety of constituent compounds in typical OSPW samples do not reach equilibrium between the sample and the SPME fiber. For illustration, the partitioning characteristics of two model naphthenic acid compounds can be considered and compared to the degree to which polycyclic aromatic hydrocarbons (PAHs) with similar log octanol–water partition coefficients (*K*_OW_) reach equilibrium with the PDMS polymer coating using the same extraction conditions (Letinski et al., 2014; Supporting Information, Figure S2). 3,5‐Dimethyladamantane‐1‐acetic acid (CAS no. 14202‐14‐3) has a predicted log *D* of 1.6 at neutral pH and a log *K*_OW_ of 4.1 under acidic conditions. It maps closest to the PAH methylfluorene (log *K*_OW_ 4.4), which reaches equilibrium within 100 min of extraction. In contrast, the more hydrophobic trans‐4'‐propyl‐(1,1'‐bicyclohexyl)‐4‐carboxylic acid (CAS no. 65355‐32‐0) has a calculated log *D* of 2.9 at neutral pH, with the protonated form having a log *K*_OW_ of 5.9 under acidic conditions. Similarly, chrysene, a four‐ring PAH, has a log *K*_OW_ of 5.8 yet reaches only 54% of equilibrium after 100 min. The BE‐SPME method somewhat mitigates this lack of equilibrium of higher‐*K*_OW_ compounds in a number of functional aspects. The automated method precisely controls the extraction parameters, so, despite being potentially far from equilibrium, high‐*K*_OW_ compounds are reproducibly extracted. The lag associated with highly hydrophobic compounds reaching equilibrium may actually mimic the uptake of these compounds in aquatic organisms, especially in acute exposures. Finally, the highest‐*K*_OW_ compounds have very low aqueous solubility which the relatively insensitive SPME GC‐FID analysis may not even detect compared to targeted mass spectrometric methods for specific compounds.

Another potential source of interlaboratory variability, common across the automated and manual versions, is the calibration procedure. Unlike conventional analytical methods where both standards and samples are introduced into the GC using the same technique (e.g., liquid injection, SPME, headspace, thermal desorption), the BE‐SPME method requires that liquid (solvent‐based) injections be made to establish the GC‐FID response factor for the 2,3‐dimethylnaphthalene calibrant to quantify the on‐fiber mass thermally desorbed from the SPME fiber. This indirect means of calibration is in contrast to conventional analysis, where any systemic biases are negated because both standards and samples are introduced using a common technique, which is not the case with the BE‐SPME method. The BE‐SPME method requires optimization of the standard injections to maximize standard response. Less than optimal standard injections result in a lower standard response, which in turn manifests in greater reported sample amounts. Standard injections incur their own set of variables including injection speed, volume injected, and the i.d. of the liner. A narrow liner i.d. is required for efficient SPME injections but lacks sufficient volume for solvent‐based injections because it cannot accommodate the vapor cloud associated with solvent expansion.

Applying the BE‐SPME method as described in the Supporting Information results in an estimated practical quantitation limit (PQL) of approximately 0.5 µmol as 2,3‐dimethylnaphthalene/ml PDMS. The PQL is derived from the lowest on‐column standard used for calibration (0.06 nmol) normalized by the PDMS volume (0.132 µl) on a 30‐µm fiber However, this PQL should only be used as a guide owing to the indirect calibration of the method and the varying chromatographic profiles of the SPMEs dependent on the source of dissolved hydrocarbons.

The development of an appropriate quality control (QC) sample for the BE‐SPME method was also briefly explored but resulted in an unsatisfactory outcome. An attempt was made to use a commercial naphthenic acid mixture, having each of the laboratories prepare aqueous solutions at a single concentration and then apply the BE‐SPME method. Unfortunately, this effort resulted in very large variability across all laboratories, even those applying the automated method (results not reported). The commercial naphthenic acid mixture is not readily soluble in water, so either lengthy mixing time is needed or dilutions must first be made in a water‐miscible solvent (e.g., methanol, acetone) for spiking into water. A field‐sourced OSPW was considered as a QC sample, but the long‐term stability of OSPWs is unknown, even under refrigerated conditions. Also considered as a BE‐SPME QC sample was a WAF of refined petroleum product such as the CGO used in the present study. However, the hydrocarbon components associated with this product class are fairly volatile. It is unlikely that its concentration in water would be maintained during extended storage, a prerequisite for a suitable QC sample.

## CONCLUSIONS

Application of the BE‐SPME method across the subset of laboratories using the automated method resulted in good agreement of results across a range of sample types. By comparison, application of the manual technique resulted in greater variability both within the individual laboratories and across laboratories. The main issue appears to be associated with the variability of mixing (type and speed) during the fiber–sample equilibration when applying the manual technique. It is important to note that equilibrium is not necessarily reached between the complex range of dissolved hydrocarbons and the SPME fiber using the automated BE‐SPME method. Neutral hydrocarbons and protonated forms of organic acids with lower *K*_OW_ (i.e., <5.1) are likely to be near equilibrium, while more hydrophobic compounds are further from equilibrium. Ultimately the automated extraction conditions provide an acceptable compromise that captures potential acute aquatic toxicity of dissolved hydrocarbons and efficient sample throughput necessary for a useful screening analysis. The BE‐SPME method reduces the complex mixture of hydrocarbons, both neutral and ionic, to a single, operationally defined exposure metric without the need to resort to complex, specific analytical methods. Application of the manual version of the BE‐SPME method has some utility as a screening tool by individual laboratories, but comparison of results to existing aquatic toxicity data sets or in meeting receiving water quality standards is limited. It is, however, equivocal whether BE‐SPME can be used to establish water quality criteria. This is partly related to the low levels (<5 BE units) that would need to be achieved and the inherently greater analytical variability at these low levels. The BE‐SPME method does not capture a range of other potential chemical and physical aquatic toxicants including metals, salts, inorganic ions, and dissolved and suspended solids.

The automated method also has an advantage regarding sample throughput compared to the manual application. In addition, the BE‐SPME may be employed in environmental forensics in sourcing hydrocarbon contamination as being from either neutral sources (e.g., crude oil, refined products) or OSPW (in the form of naphthenic acids).

Ultimately, this round robin test program confirmed that BE‐SPME is very much an operationally defined method. Harmonization across all of the method's parameters is critical to make effective interlaboratory comparisons, especially when used to predict aquatic toxicity.

## Supporting Information

The Supporting Information is available on the Wiley Online Library at https://doi.org/10.1002/etc.5340.

## Disclaimer

Daniel Letinski, Asfaw Bekele, and Martin Connelly are employees of affiliate companies of Exxon Mobil Corporation. ExxonMobil and its affiliates, including Imperial, produce materials and waste streams that are represented by the ones reported in this research. Imperial is a sustaining member of the Canadian Oils Sands Innovation Alliance.

## Author Contribution Statement

**Daniel J. Letinski**: Conceptualization; Formal analysis; Methodology; Investigation; Supervision; Writing—original draft. **Asfaw Bekele**: Funding acquisition; Project administration, Writing—review and editing. **Martin J. Connelly**: Methodology; Resources; Data curation.

## Supporting information

This article includes online‐only Supporting Information.

Supporting information.

## Data Availability

Data, associated metadata, and calculation tools are available from the corresponding author in order to maintain the anonymized identities of the participating labs. (daniel.j.letinski@exxonmobil.com).
