# Supplementary material for: Interlaboratory Comparison of a Biomimetic Extraction Method Applied to Oil Sands Process–Affected Waters
Source: Environ Toxicol Chem. 2022 Jun 25;41(7):1613–22. doi: 10.1002/etc.5340 (PMC9328283; doi:10.1002/etc.5340)
Supplement: Supplementary file 1 — Supporting information. [file ETC-41-1613-s001.docx]

**Supporting Information** *BE-SPME Protocol for Hydrocarbons in Water including Oil Sands Process-Affected Waters* Summary This method describes a SPME GC-FID method for the quantification of bioavailable, dissolved hydrocarbons for both neutral and ionizable organics including acid extractable organics (AEO) in OSPWs. Consumables • 20 mL glass vials with Teflon faced septum screw or crimp caps (metallic caps should be used for use with SPME autosamplers) • 30 µm PDMS SPME fibers (Supelco/Sigma Aldrich) • Sigma Aldrich Certified Reference Material- Oil Sand Process-Affected Water Calibration Standard Kit containing 2,3 dimethylnaphthalene calibrant. Product ID: RTC000200-2ML • 5 µL GC syringes • Phosphoric acid (H_3_PO_4_ 85%, ACS grade) Instrumentation • Gas chromatograph equipped with a flame ionization detector (GC-FID) and a 15 m x 0.53 with 1.5 µm Rtx-1 stationary phase (Restek) or equivalent. • GC capillary inlet configured for splitless injections and equipped with a narrow (≤ 1 mm id) liner with no glass wool or packing to accommodate SPME fiber injections. • A CTC Analytics “rail” based GC autosampler (or equivalent) capable of automated SPME injections and sample-fiber agitation. • Chromatographic Data System interfaced with the GC for acquisition, processing,calibration and reporting of BE-SPME results. The data system must be able to integrate broad, unresolved “area-under-the curve” chromatograms. Ideally, the data system will also have the capability to perform digital subtraction of background GC baselines from sample chromatograms.

Sample Preparation Water samples are either collected in, or transferred to, ca. 20 mL glass vials with Teflon caps and filled so there is no headspace. If analysis of both neutral and acid-extractable organics is required, samples are acidified with 50 µL of phosphoric acid bringing the sample pH to approximately 2.0 to 2.4. If only neutral dissolved hydrocarbons are expected to be present, sample acidification is not required. Samples are then transferred to the SPME autosampler. Solid Phase Microextraction Samples are equilibrated with the 30 µm PDMS SPME fibers for 100 minutes with orbital agitation (250 rpm) at 30^o^C in the autosampler’s incubator module. Following completion of the equilibration/extraction period, the autosampler automatically retracts the fiber and injects it into the GC injection port at 280^o^C. The fiber is thermally desorbed in the injection port for three minutes to fully desorb the organic components that partitioned into the PDMS. Use of a SPME autosampler permits a single fiber to be used for multiple, successive sample extractions. Alternatively, if an SPME autosampler is not available, the SPME-OSPW sample extraction can be performed manually. In this case, the samples are prepared as described above but each sample should also include a Teflon micro stir-bar. The SPME fiber assembly is manually inserted through the septum cap and the fiber extended exposing it to the water sample. The vial containing sample, stir bar and exposed SPME fiber is placed on a stir plate where it is rapidly mixed for 100 minutes. At the end of the mixing time, the fiber is retracted, the SPME assembly removed and the assembly is affixed to a SPME holder. The fiber can then be manually injected/thermally desorbed for three minutes in the GC injection port as described above.

GC Conditions The carrier gas is helium at a constant flow rate of 17 mL/min. The GC oven is temperature programed from 40^o^C for three minutes up to 300^o^C at a rate of 45^o^C/minute. The FID temperature is 300^o^C and inlet temperature is 280^o^C. The detector signal attenuation is -3 (Perkin Elmer Autosystem GC).

Calibration and Results Processing The method is calibrated by making 0.5 µL liquid (solvent) injections using an air-gap (0.5 µL) technique. A series of aromatic hydrocarbon standards (toluene, o-xylene, 2-methylnaphthalene, 2,3 dimethylnaphthalene and 9-methylantracene) in dichloromethane are manually injected. The instrument conditions are exactly the same as those used to analyze the SPME fiber extracts except that the splitless injection split time is reduced to one minute to accommodate the solvent peak. Calibration is performed at three concentration levels (approximately 20, 100 and 200 µg/mL) corresponding to on-column amounts of approximately 0.06, 0.3 and 0.6 nanomoles. The average molar response factor of 2,3-dimethylnaphthalene is used to convert the measured GC-FID response (total integrated area) to nanomoles of organic constituents on the PDMS fiber. Where necessary, SPME sample chromatograms are digitally background corrected by subtraction with a blank GC chromatographic run, to account for column bleed. Chromatograms are acquired and processed using Perkin Elmer TotalChrom chromatographic software (or equivalent). Integration parameters are optimized specifically for each sample type to integrate the area under the curve attributable to the SPME extracted sample. As the automated SPME extraction was performed at 30^o^C, a temperature correction factor of 1.08 is applied to normalize results to a previously applied manual technique where extraction took place at room temperature (22^o^C). Temperature corrected BE-SPME results are then normalized to the volume of PDMS on the fiber and reported as micromoles (µmol) as 2,3-dimethylnaphthalene / milliliter (mL) PDMS. The detection limit for the automated SPME method is approximately 0.5 µmol as 2,3-dimethylnaphthalene / milliliter (mL) PDMS. For the automated BE-SPME application, the ratio of PDMS to water sample is approximately 6.6 x 10-6 v/v. For the older, manual method where a 1 cm, 100 µm PDMS (0.0.612 µL PDMS) SPME fiber was used on ca. 140 mL water samples, the ratio was 4.4x10-6 v/v.

Table S-1 BE-SPME Analysis Parameters

| **Lab** | **GC-FID** | **Column** | **Liner**  **ID**  **(mm)** | **Standard injection volume**  **(µL)** | **Calibration**  **(%RSD**  **or r^2^)** | **BE method** | **Agitation**  **(rpm)** |
| --- | --- | --- | --- | --- | --- | --- | --- |
| A | Agilent 7890B | 15m x 0.53mm id  1.5μm Rtx-1 (Restek) | 0.75 | 1  auto | 0.9997 | Auto | Orbital  250 |
| B | Agilent 7890B | 15m x 0.53mm id  1.5 µm Rtx-1 (Restek) with 1m deactivated pre-column and 40cm transfer capillary  (both 0.53mm) | 0.75 | 1  auto | 13% | Auto | Orbital  250 |
| E | Perkin Elmer Autosystem XL | 15m x 0.53mm id  1.5μm Rtx-1 (Restek) | 1 | 0.5  manual | 4.1% | Auto | Orbital  250 |
| I | Thermo Trace 1310 | 15m x 0.53mm id  1.8μm Rtx-1 (Restek) | 2 | 0.5  manual | 0.99995 | Auto | Orbital  250 |
| N | Agilent 7890A | Rtx-1 | 2 | 0.5  manual |  | Auto | Orbital  250 |
| Q | Agilent 7890B | 15m x 0.53mm id x 1.5 µm VF-1MS (Agilent J&W) | 1 | 1  auto | 0.999 | Auto | Orbital  250 |
| F | HP 5890 series II | 15m x 0.53mm id  1.5 µm DB-1 (Agilent J&W) | 0.75 | 0.5  manual | 0.9835 - 0.9999 | Manual | Stir  250-400 |
| H | Agilent 7890 | 15m x 0.53mm x 3.0µm DB-1 Agilent J&W) | 2 | 0.5  manual | 0.997323 | Manual | Stir |
| V | Varian 450 | 15m x 0.53mm id  1.5μm Rtx-1 (Restek) | 2 | 0.5  manual | 9.2% | Manual | Stir |
| M | Agilent 7890B | 15 m, 0.53 mm, 1.50 µm DB-1 (Agilent J&W) | 2 | 1  manual | 10% | Manual | Stir |

The results for the automated and manual procedures are reported separately because the variances from the two procedures are significantly different according to the Levene’s test at the 5% level of significance (Figure S1A). Levene’s test was conducted using mean BE-SPME values from each participating laboratory using the Mintab software (Version 17). Result from Levene’s test for the equality of variances between the two procedures for the acidified samples corresponding to June 2019 OSPW and July 2019 OSPW are shown in Figure S1A. The box plots further demonstrate the differences in variability of the automated versus manual procedures (Figure S1B).


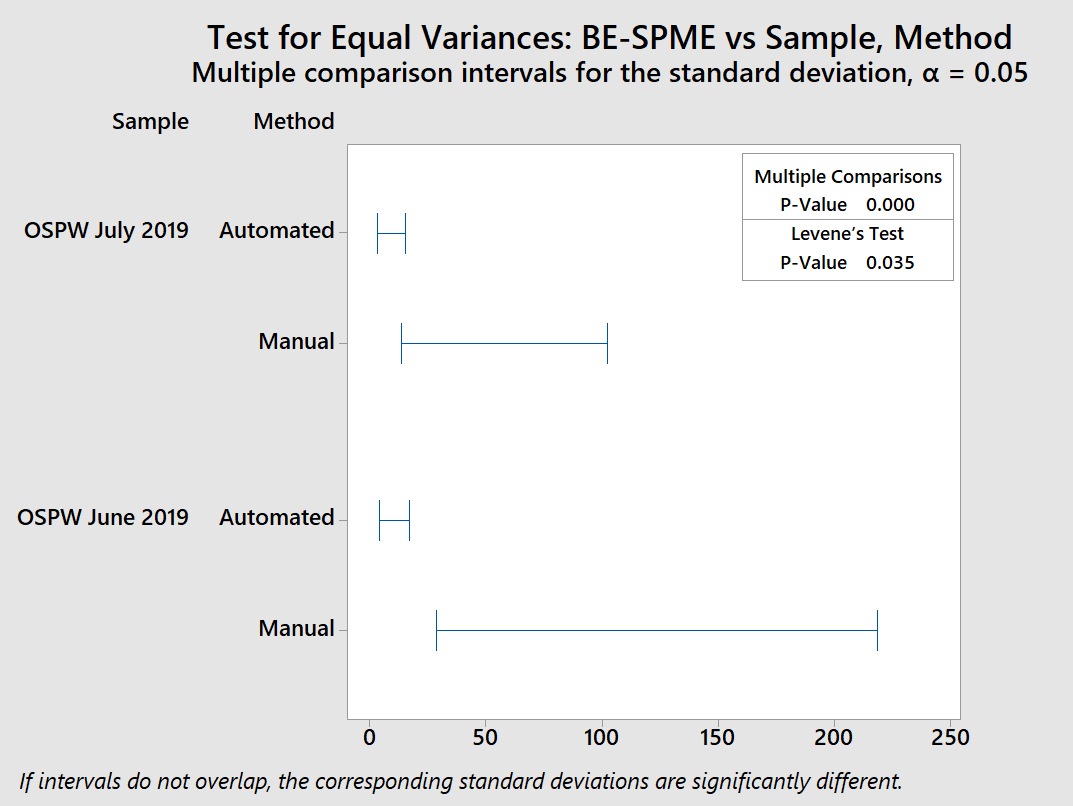


Figure S1A. Result of test of equal variances. In Levene’s test, the null hypothesis is that all variances are equal. A P-value of 0.035 for Levene’s test is evidence for rejecting the null hypothesis. Further, if the graphical multiple comparison intervals for the standard deviations do not overlap, as is the case here for the two methods, the corresponding standard deviations are significantly different at 5% probability.


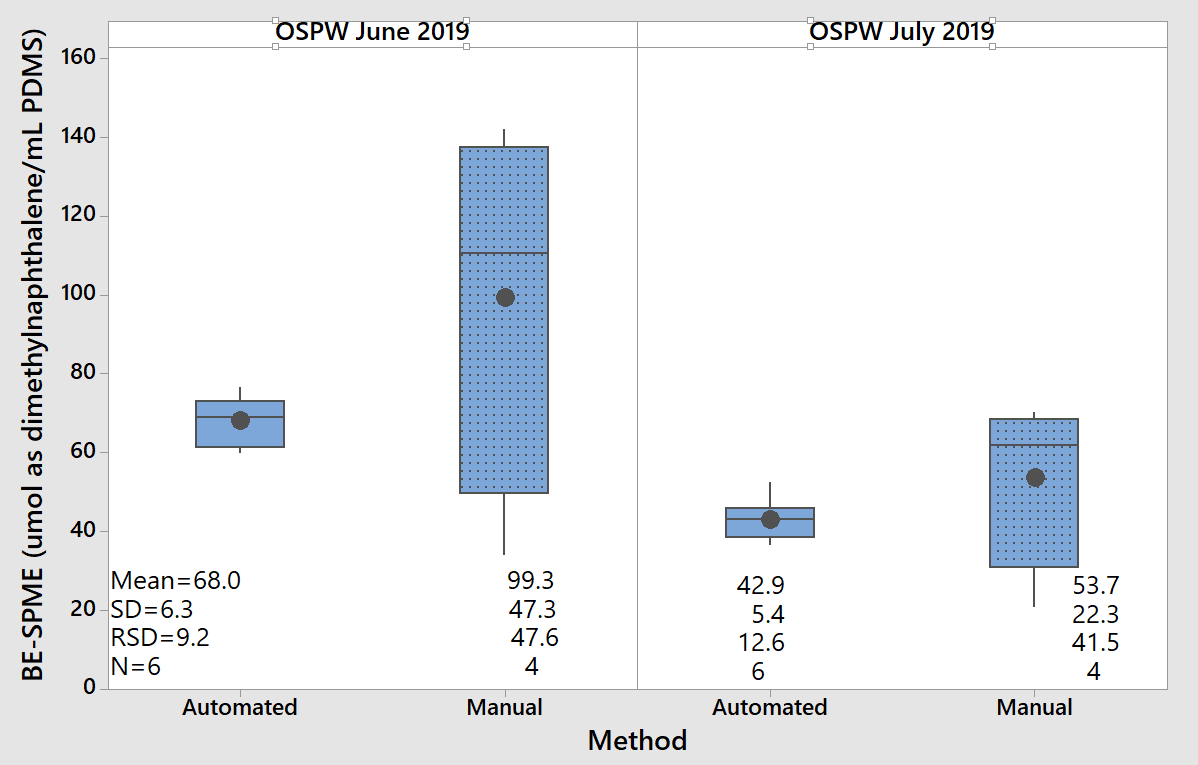


Figure S1B. Box and whisker plots showing the variability in BE-SPME measurements using the automated and manual methods for acidified samples from June 2019 OSPW and July 2019 OSPW. The horizontal line in the middle represents the median, the black dot represents the mean.

Figure S2. Log K_ow_ versus Equilibrium after 100 minutes of SPME Extraction of PAHs. Each compound spiked in water at concentration of 0.5 ng/mL in ca. 20 mL vials. Replicate vials extracted using either 30 µm (0.132 µL PDMS) or 7 µm (0.028 µL PDMS) SPME fibers with 250 rpm orbital agitation at 30^o^C after 50, 100, 300 and 600 minutes. Analysis performed on a Varian Saturn 2000 Ion Trap GC-MS. Equilibrium assumed to have been reached at 600 minutes for reference.
